# Sulbactam-durlobactam in combination with aztreonam and carbapenems against carbapenem-resistant *Acinetobacter baumannii*: an assessment using the MIC-based broth disk elution

**DOI:** 10.1128/jcm.00709-25

**Published:** 2025-07-24

**Authors:** C. Koenig, D. P. Nicolau, T.E. Asempa

**Affiliations:** 1 Center for Anti-Infective Research and Development, Hartford Hospital 23893, Hartford, Connecticut, USA; 2 Department of Intensive Care Medicine, University Hospital Hamburg-Eppendorf, Hamburg, Germany; 3 Division of Infectious Diseases, Hartford Hospital 23893, Hartford, Connecticut, USA; Marquette University, Milwaukee, Wisconsin, USA

**Keywords:** sulbactam-durlobactam, *Acinetobacter baumannii*, CRAB

## LETTER

Sulbactam-durlobactam (SUD) resistance among Acinetobacter baumannii-*calcoaceticus* complex (ABC) is rare (2%–3%) and likely due to the interplay of metallo-β-lactamase (MBL) production, mutations in PBP3, and increased efflux activity (1–5). The combination of SUD plus aztreonam (MBL-stable β-lactam) or SUD plus carbapenem could potentially overcome the aforementioned resistance mechanisms and be considered an additional option in these scenarios (6–8). Recently, a broth disk elution (BDE) test was CLSI-approved to determine susceptibility to the ceftazidime-avibactam-aztreonam combination (9, 10). Therefore, we aimed to evaluate the activity of SUD plus aztreonam (ATM), imipenem (IPM), or meropenem (MEM) using the BDE method against ABC.

Twenty-four clinical ABC isolates were utilized and included 20 carbapenem-resistant isolates. Of these isolates, 6 were SUD-susceptible (MIC ≤ 4 µg/mL), 11 were SUD-intermediate (MIC 8 µg/mL), and 7 were SUD-resistant (MIC ≥ 16 µg/mL) by broth microdilution testing (Table 1) (10). Isolates with a SUD MIC ≥ 8 µg/mL underwent whole-genome sequencing as previously described and revealed New Delhi MBL genes (*bla*_NDM_) in seven isolates (4).

**TABLE 1:** Categorical results (turbidity/haziness: “+”; no growth: “−“) of the broth disk elution assay for *Acinetobacter baumannii* complex isolates^*b*^

Isolate ID	SUD MIC (µg/mL)	SUD	ATM	ATM/SUD	IPM	IPM/SUD	MEM	MEM/SUD	MIC (µg/mL)		
									ATM	IPM	MEM
*A. baumannii* NCTC 13304	0.5	−	+	−	+	−	+	−	64	32	64
*Escherichia coli* ATCC 25922	≤0.12	−	−	−	−	−	−	−	0.25	0.12	0.12
*Escherichia coli* AR Bank #0348	128	+	+	+	+	+	+	+	>64	>32	32
CRAB 315	0.5	−	+	−	+	−	+	−	>64	>32	64
ACNB 1111	0.5	−	+	−	−	−	−	−	64	0.25	0.25
ACNB 1732	0.5	−	+	−	−	−	−	−	32	0.25	0.25
CRAB 320	1	−	+	−	+	−	+	−	64	32	32
CRAB 117	1	−	+	−	+	−	+	−	64	>32	64
ACNB 1360	1	−	+	−	−	−	−	−	16	0.25	0.5
CRAB 1716	8	−	+	−	+	−	+	−	>64	>32	>64
CRAB 326	8	−	+	−	+	−	+	−	>64	32	64
CRAB 341	8	−	+	−	+	−	+	−	>64	>32	64
CRAB 513	8	+	+	+	+	−	+	+	64	8	16
CRAB 133	8	−	+	−	+	−	+	−	>64	>32	>64
CRAB 1336	8	−	+	−	+	−	+	−	>64	>32	>64
CRAB 1433	8	−	+	−	+	−	+	−	>64	32	64
ACNB 211	8	−	−	−	−	−	−	−	0.12	2	0.06
CRAB 2114	8	−	+	−	+	−	+	−	>64	>32	>64
CRAB 2119^*a*^	8	−	+	−	+	−	+	−	64	>32	>64
CRAB 2122^*a*^	8	−	+	−	+	−	+	−	64	>32	>64
CRAB 124	16	−	+	−	+	−	+	−	>64	>32	>64
CRAB 134	16	−	+	−	+	−	+	−	>64	32	64
CRAB 622^*a*^	32	+	+	+	+	+	+	+	64	>32	>64
CRAB 621^*a*^	64	+	+	+	+	+	+	+	>64	>32	>64
CRAB 167^*a*^	64	+	+	+	+	+	+	+	>64	>32	>64
CRAB 325^*a*^	128	+	+	+	+	+	+	+	64	>32	>64
CRAB 332^*a*^	128	+	+	+	+	+	+	+	64	>32	>64

^*a*^ Isolate positive for blaNDM.

^*b*^ SUD, sulbactam-durlobactam; ATM, aztreonam; IPM, imipenem; MEM, meropenem.

Briefly, SUD 10/10 µg, ATM 30 µg, IPM 10 µg, and MEM 10 µg disks (Hardy Diagnostics, CA) were added to separate 5 mL cation-adjusted Mueller-Hinton broth (Becton Dickinson, NJ) tubes in the following combinations: no disk (growth control), 2× SUD (two disks), ATM, ATM/2× SUD, IPM, IPM/2× SUD, MEM, MEM/2× SUD, and incubated for 30–60 min to allow for drug diffusion from the disk(s). Disk-content/broth ratio allowed for final SUD, ATM, IPM, and MEM concentrations of 4/4, 6, 2, and 2 µg/mL, respectively. Aliquots (25 µL) of the 0.5 McFarland culture were added to each tube, vortexed, incubated at 35^o^C ± 2^o^C for 20 h, and visibly assessed for growth (haziness/turbidity: not susceptible) or no growth (susceptible). Quality control strains were tested with each run (Table 1, Fig. S1), and BDE testing was repeated for confirmation if discordance between MIC susceptibility category and BDE result was observed.

*SUD-susceptible ABC (n=6)*. The BDE method was concordant with MIC across all single-agent tubes. As expected, SUD-combination tubes exhibited no growth given SUD susceptibility.

**Fig 1:**
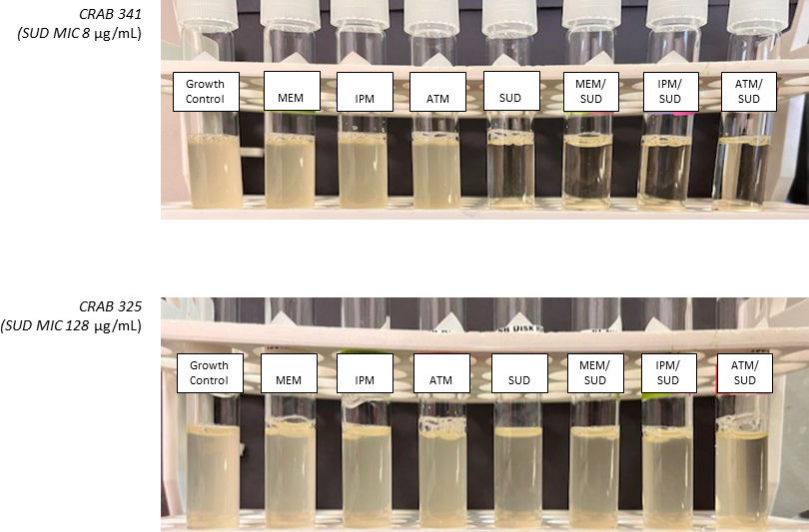
Representative BDE results for two isolates: CRAB 341 (SUD MIC of 8 µg/mL; intermediate) and CRAB 325 (SUD MIC of 128 µg/mL; resistant).

*SUD-intermediate ABC (n=11)*. No growth was observed in 10/11 tubes despite SUD MIC of 8 µg/mL, including 2 NDM-positive isolates (Fig. 1). Of note, one isolate (CRAB 513, negative for *bla*_NDM_) demonstrated bacterial growth in the SUD monotherapy tube, but no growth when combined with IPM (MIC 8 µg/mL), likely reflecting potential for synergy against isolates with low-level resistance to both agents.

*SUD-resistant ABC (n=7)*. Among the two isolates with SUD MIC 16 µg/mL, no growth was observed in both SUD-monotherapy tubes and SUD-combination tubes. Among the five isolates with MIC ≥32 µg/mL, including five NDM-positive strains, we observed bacterial growth in all SUD-monotherapy and SUD-combination tubes (Fig. 1).

ABC resistance to SUD is rare (2%–3%); thus, the study size of this SUD non-susceptible data set is a strength. No antagonism was observed. Replicate testing and CLSI quality control tests, including the addition of an *A. baumannii*-specific QC (*A. baumannii* NCTC 13304), were performed (Table S1). A limitation of the present study is that broth microdilution MICs of the antimicrobial combinations were not performed to compare with BDE combination tube results. A larger multicenter study utilizing different broth and disk manufacturers is required to confirm the accuracy and reproducibility of these BDE results, given test performance variability, especially against clinical isolates with antimicrobial MICs that shoulder the breakpoint (9–11). Lastly, the concentration of antimicrobials eluted from the disks in broth was assumed and not confirmed. Notably, these FDA-approved antimicrobial disks are optimized for drug elution on agar, while disk elution in broth is experimental and thus needs confirmation, including the extent of drug elution from the 2 SUD disks/tube necessary for this study.

In summary, SUD susceptibility in the monotherapy tubes was observed with the BDE method for isolates with SUD MICs of ≤16 µg/mL. This drove susceptibility in the corresponding SUD-combination tubes. Bacterial growth was observed in SUD-monotherapy and combination tubes against all isolates with SUD MICs of ≥32 µg/mL (all NDM+).

## SUPPLEMENTAL MATERIAL

Supplemental tables and figure jcm.00709-25-s0001.docx Tables S1 and S2, and Fig. S1.

ASM does not own the copyrights to Supplemental Material that may be linked to, or accessed through, an article. The authors have granted ASM a non-exclusive, world-wide license to publish the Supplemental Material files. Please contact the corresponding author directly for reuse.
