# Supplementary material for: Sulbactam-durlobactam in combination with aztreonam and carbapenems against carbapenem-resistant Acinetobacter baumannii: an assessment using the MIC-based broth disk elution
Source: J Clin Microbiol. 2025 Jul 24;63(8):e00709-25. doi: 10.1128/jcm.00709-25 (PMC12345227; doi:10.1128/jcm.00709-25)
Supplement: Supplemental tables and figure — Tables S1 and S2, and Fig. S1. [file jcm.00709-25-s0001.docx]

**Supplement**

**Sulbactam-Durlobactam in combination with Aztreonam and Carbapenems against Carbapenem-Resistant *Acinetobacter baumannii* clinical isolates: An Assessment using the MIC-based Broth Disk Elution**

C. Koenig^1,2^, D.P. Nicolau^1,3^, T.E. Asempa^1^

^1^Center for Anti-Infective Research and Development, Hartford Hospital, Hartford, Connecticut, USA

^2^Department of Intensive Care Medicine, University Hospital Hamburg-Eppendorf, Hamburg, Germany

^3^ Division of Infectious Diseases, Hartford Hospital, CT, USA

Table S1. Broth MIC and BDE individual results (turbidity/haziness: “+”; no growth: “-“) for quality control strains and clinical ABC isolates. A priori replicate testing performed for all SUD-resistant isolates, select SUD-intermediate and -susceptible isolates. Repeat testing performed for all isolates with discordant BDE-MIC results

| **QC Strain and clinical isolates** | **SUD MIC (µg/mL)** | **SUD** | **ATM** | **ATM/**  **SUD** | **IPM** | **IPM/**  **SUD** | **MEM** | **MEM/**  **SUD** | **MIC (µg/mL)** | | |
| --- | --- | --- | --- | --- | --- | --- | --- | --- | --- | --- | --- |
|  |  |  |  |  |  |  |  |  | **ATM** | **IPM** | **MEM** |
| *A. baumannii* NCTC 13304 | 0.5 | -/-/-/-/-/- | +/+/+/+/+/+ | -/-/-/-/-/- | +/+/+/+/+/+ | -/-/-/-/-/- | +/+/+/+/+/+ | -/-/-/-/-/- | 64 | 32 | 64 |
| *Escherichia coli* ATCC 25922 | ≤0.12 | -/-/-/-/-/- | -/-/-/-/-/- | -/-/-/-/-/- | -/-/-/-/-/- | -/-/-/-/-/- | -/-/-/-/-/- | -/-/-/-/-/- | 0.25 | 0.12 | 0.12 |
| *Escherichia coli* AR Bank #0348 | 128 | +/+/+/+/+/+ | +/+/+/+/+/+ | +/+/+/+/+/+ | +/+/+/+/+/+ | +/+/+/+/+/+ | +/+/+/+/+/+ | +/+/+/+/+/+ | >64 | >32 | 32 |
| CRAB 315 | 0.5 | -/-/-/- | +/+/+/+ | -/-/-/- | +/+/+/+ | -/-/-/- | +/+/+/+ | -/-/-/- | >64 | >32 | 64 |
| ACNB 1111 | 0.5 | -/-/- | +/+/+ | -/-/- | -/-/- | -/-/- | -/-/- | -/-/- | 64 | 0.25 | 0.25 |
| ACNB 1732 | 0.5 | - | + | - | - | - | - | - | 32 | 0.25 | 0.25 |
| CRAB 320 | 1 | - | + | - | + | - | + | - | 64 | 32 | 32 |
| CRAB 117 | 1 | -/- | +/+ | -/- | +/+ | -/- | +/+ | -/- | 64 | >32 | 64 |
| ACNB 1360 | 1 | - | + | - | - | - | - | - | 16 | 0.25 | 0.5 |
| CRAB 1716 | 8 | -/- | +/+ | -/- | +/+ | -/- | +/+ | -/- | >64 | >32 | >64 |
| CRAB 326 | 8 | - | + | - | + | - | + | - | >64 | 32 | 64 |
| CRAB 341 | 8 | - | + | - | + | - | + | - | >64 | >32 | 64 |
| CRAB 513 | 8 | +/+/+ | +/+/+ | -/+/+ | +/+/+ | -/-/- | +/+/+ | -/+/+ | 64 | 8 | 16 |
| CRAB 133 | 8 | - | + | - | + | - | + | - | >64 | >32 | >64 |
| CRAB 1336 | 8 | -/- | +/+ | -/- | +/+ | -/- | +/+ | -/- | >64 | >32 | >64 |
| CRAB 1433 | 8 | - | + | - | + | - | + | - | >64 | 32 | 64 |
| ACNB 211 | 8 | -/- | -/- | -/- | -/- | -/- | -/- | -/- | 0.12 | 2 | 0.06 |
| CRAB 2114 | 8 | -/-/+/-/- | +/+/+/+/+ | -/+/+/-/- | +/+/+/+/+ | -/+/+/-/- | -/+/+/+/+ | -/+/+/-/- | >64 | >32 | >64 |
| CRAB 2119 | 8 | -/- | +/+ | -/- | +/+ | -/- | +/+ | -/- | 64 | >32 | >64 |
| CRAB 2122 | 8 | -/-/- | +/+/+ | +/-/- | +/+/+ | +/-/- | +/+/+ | -/-/- | 64 | >32 | >64 |
| CRAB 124 | 16 | -/-/- | +/+/+ | -/-/- | +/+/+ | -/-/- | +/+/+ | -/-/- | >64 | >32 | >64 |
| CRAB 134 | 16 | -/-/- | -/+/+ | -/-/- | +/+/+ | -/-/- | +/+/+ | -/+/- | >64 | 32 | 64 |
| CRAB 622 | 64 | +/+ | +/+ | +/+ | +/+ | +/+ | +/+ | +/+ | 64 | >32 | >64 |
| CRAB 621 | >64 | -/+/+ | +/+/+ | -/+/+ | +/+/+ | -/+/+ | +/+/+ | -/+/+ | >64 | >32 | >64 |
| CRAB 167 | >64 | +/+ | +/+ | +/+ | +/+ | +/+ | +/+ | +/+ | >64 | >32 | >64 |
| CRAB 325 | 64 | +/+ | +/+ | +/+ | +/+ | +/+ | +/+ | +/+ | 64 | >32 | >64 |
| CRAB 332 | 64 | +/+ | +/+ | +/+ | +/+ | +/+ | +/+ | +/+ | 64 | >32 | >64 |

ATM, aztreonam; IPM, imipenem; MEM, meropenem; SUD, sulbactam-durlobactam

Figure S1. BDE results for the three quality control strains


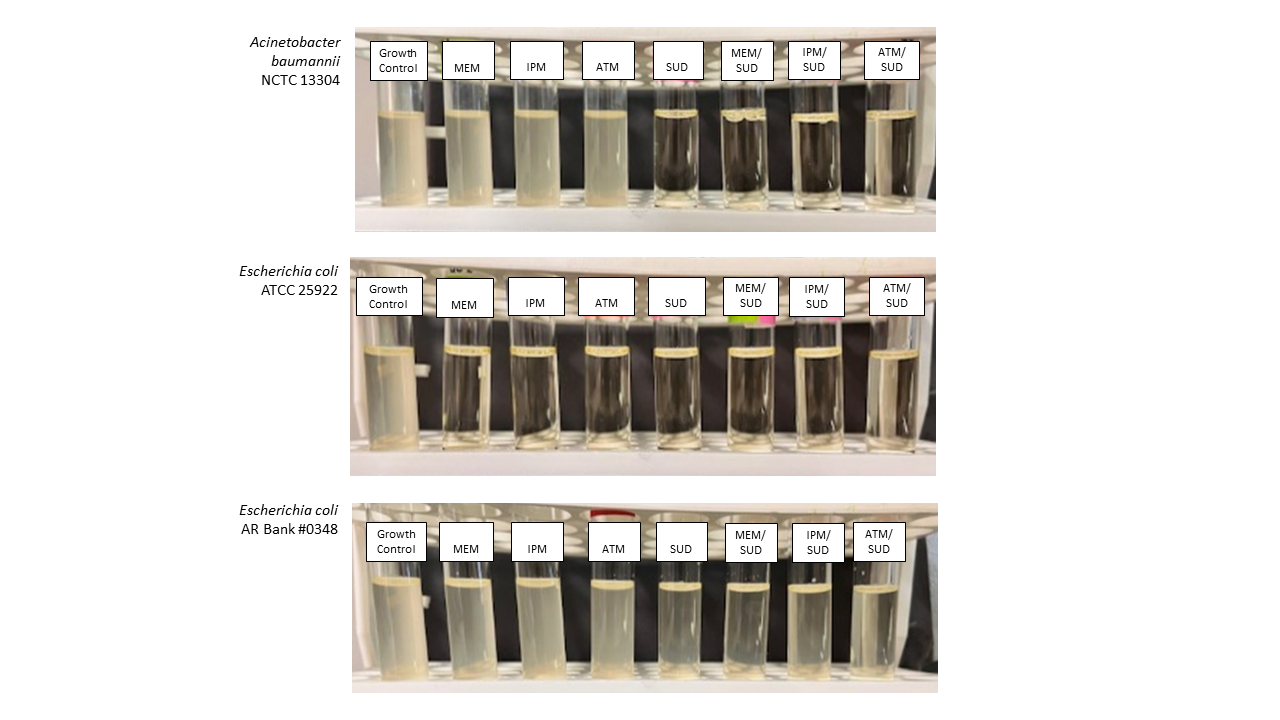


ATM, aztreonam; IPM, imipenem; MEM, meropenem; SUD, sulbactam-durlobactam

Table S2. Utilized broth and disks

| **Item** | **Manufacturer** | **Lot No.** |
| --- | --- | --- |
| Cation-adjusted Mueller-Hinton broth | Becton, Dickinson, NJ, USA | 4102223 |
| Sulbactam/durlobactam 10/10µg disk | Hardy Diagnostics, CA, USA | 508538 |
| ATM 30µg disk | Hardy Diagnostics, CA, USA | 2244457 |
| IPM 10µg disk | Hardy Diagnostics, CA, USA | 500182, 3214950 |
| MEM 10µg disk | Hardy Diagnostics CA, USA | 4120704 |
